# Microbial resolution of whole genome shotgun and 16S amplicon metagenomic sequencing using publicly available NEON data

**DOI:** 10.1371/journal.pone.0228899

**Published:** 2020-02-13

**Authors:** Kyle D. Brumfield, Anwar Huq, Rita R. Colwell, James L. Olds, Menu B. Leddy

**Affiliations:** 1 Maryland Pathogen Research Institute, University of Maryland, College Park, Maryland, United States of America; 2 University of Maryland Institute for Advanced Computer Studies, University of Maryland, College Park, Maryland, United States of America; 3 CosmosID Inc., Rockville, MD, United States of America; 4 Schar School, George Mason University, Arlington, Virginia, United States of America; 5 Essential Environmental and Engineering Systems, Huntington Beach, California, United States of America; University of Illinois College of Medicine, UNITED STATES

## Abstract

Microorganisms are ubiquitous in the biosphere, playing a crucial role in both biogeochemistry of the planet and human health. However, identifying these microorganisms and defining their function are challenging. Widely used approaches in comparative metagenomics, 16S amplicon sequencing and whole genome shotgun sequencing (WGS), have provided access to DNA sequencing analysis to identify microorganisms and evaluate diversity and abundance in various environments. However, advances in parallel high-throughput DNA sequencing in the past decade have introduced major hurdles, namely standardization of methods, data storage, reproducible interoperability of results, and data sharing. The National Ecological Observatory Network (NEON), established by the National Science Foundation, enables all researchers to address queries on a regional to continental scale around a variety of environmental challenges and provide high-quality, integrated, and standardized data from field sites across the U.S. As the amount of metagenomic data continues to grow, standardized procedures that allow results across projects to be assessed and compared is becoming increasingly important in the field of metagenomics. We demonstrate the feasibility of using publicly available NEON soil metagenomic sequencing datasets in combination with open access Metagenomics Rapid Annotation using the Subsystem Technology (MG-RAST) server to illustrate advantages of WGS compared to 16S amplicon sequencing. Four WGS and four 16S amplicon sequence datasets, from surface soil samples prepared by NEON investigators, were selected for comparison, using standardized protocols collected at the same locations in Colorado between April-July 2014. The dominant bacterial phyla detected across samples agreed between sequencing methodologies. However, WGS yielded greater microbial resolution, increased accuracy, and allowed identification of more genera of bacteria, archaea, viruses, and eukaryota, and putative functional genes that would have gone undetected using 16S amplicon sequencing. NEON open data will be useful for future studies characterizing and quantifying complex ecological processes associated with changing aquatic and terrestrial ecosystems.

## Introduction

Over the past decade, interest in total microbial community composition and dynamics of complex environments has increased significantly. This is because the estimated total number of microbial cells in the earth’s biosphere exceeds 10^30^ [1], and the microbes themselves harbor potentially up to an additional 10^31^ phages [2]. All of which have helped shape the planet and its biosphere [3]. Diverse microbial communities flourish in a wide spectrum of complex environments ranging from the human gut [4], rhizosphere [5], and conventionally inhospitable habitats, such as geothermal hot springs [6] and Antarctic volcano mineral soils [7]. Furthermore, microbial activities play a critical role in the biogeochemistry of the planet [8,9] and wellbeing of macroorganisms [10].

Traditionally, microbial communities have been defined using culture dependent methods to detect and enumerate microorganisms. However, it is estimated that the vast majority of prokaryotic genospecies remain uncultured [11], and genomes of uncultured microorganisms encode a largely untapped reservoir of novel metabolites and metabolic processes [12]. Accordingly, the field of metagenomics has developed rapidly and effectively obviates the need to isolate and culture microorganisms by utilizing the genetic material of a sample to identify accurately the functional gene composition [12,13]. That major accomplishment has allowed in depth comparison and exploration of microbial ecology [14,15], including the metabolic profile of complex microbial ecosystems [16,17].

Since the emergence of metagenomics, where DNA is sequenced directly from environmental samples, sequencing for microbial identification has evolved to include a variety of approaches. The polymerase chain reaction (PCR) is one of the fundamental methods currently being used for taxonomic identification, commonly employing amplification of variant regions in macromolecules conserved across species [18]. PCR-based metagenomics is now routine in gene prospecting by direct amplification of specific genes [19] or colony PCR to screen metagenomic libraries [20]. Moreover, PCR amplification of specific genes is used to evaluate microbial species diversity based on sequence composition. The use of 16S ribosomal RNA (rRNA) genes—that occur in one or more copies in most bacterial and archaeal genomes [21] and also present in mitochondrial genomes [22]—is widely recognized as the ‘Gold Standard’ for prokaryotic identification. The 16S rRNA gene sequencing method generally employs universal PCR primers to amplify hypervariable regions of the 16S rRNA gene that infer taxonomic identification by bioinformatic alignment against various rRNA sequence databases [23–26], such as the Ribosomal Database Project (RDP) [27], SILVA Ribosomal RNA Gene Database Project [28], or Greengenes [29] databases.

With advances being made in DNA sequencing technology, the cost of sequencing has decreased and whole genome shotgun metagenomic sequencing is attractive for many laboratories to study all of the genes in all organisms present in uncultured microbial communities in complex samples [30]. Instead of targeting specific genomic markers, total DNA is extracted and sheared into fragments that are independently sequenced and aligned, for taxonomic identification to genomic databases, such as the Reference Sequence (RefSeq) [31], GenBank [32], or Pathosystems Resource Integration Center (PATRIC) [33] databases. DNA whole genome shotgun metagenomics has also been complemented with metatranscriptomic or metaproteomic approaches to describe microbial function [34,35]. Accordingly, curated databases for genome annotation sequences, e.g., Subsystems ontology [36], and protein, e.g., SwissProt [37], have been established.

Development of the high-throughput analytical strategies has changed data handling and processing for microbiology. A single biological sample can now be processed in parallel to generate high-throughput data composed of genome sequences, gene and protein expression patterns, or metabolite fluxes, which each require unique postgenomic computer manipulation for analysis [38]. As a result, numerous efforts [38–40], specially the Science Commons Protocol for Implementing Open Access Data [41], have surfaced that strive to initiate the regulation of open-access protocols for data management and sharing. However, standardization of laboratory procedures to ensure quality and interoperability of ‘Big Data’ produced through sequencing, notably amplicon and shotgun metagenomics, remains critical [42,43]. The number of metagenomic datasets has increased dramatically, creating a need for standardized operating procedures for sample collection, processing, and data storage. It is now very important to be able to compare sample sequences to a known database, thereby providing information for subsequent analyses, including taxonomic identification and comparison [13].

One of the goals of the National Ecological Observatory Network (NEON) is to enable researchers to ask questions on a regional to continental scale that involve a variety of environmental challenges and provide high-quality, integrated, and standardized data derived from standard field sites across the United States. The NEON soil microbe metagenome and marker gene sequences projects are derived from soil microbial sampling, contain quality-controlled metadata and results for the NEON shotgun metagenomic and 16S marker gene sequences, respectively [44]. In this study, we demonstrate effective use of publicly available NEON soil metagenomic sequencing datasets hosted on the open access Metagenomics Rapid Annotation using Subsystem Technology (MG-RAST) server [45] to assess the feasibility of employing openly sourced NEON data. We compare and contrast whole genome shotgun metagenomic sequencing and 16S amplicon sequencing for application in environmental metagenomics.

## Materials and methods

### Data type and sample selection

NEON provides open access to information obtained from soil and freshwater (surface and benthic) samples on their microbial content. All microbe metagenome (Neon Data Product ID = DP1.10107.001) and marker gene (NEON Data Product ID = DP1.10108.001) sequence data were previously uploaded to the open-submission MG-RAST data portal [45] for processing and analysis by NEON investigators using standardized protocols [44]. As of August 22, 2019, the MG-RAST server, version 4.0.3, hosted 66,454 public and 390,819 total metagenomes containing 1,498 billion DNA sequences (209.08 tera base pairs).

To select samples for the investigation reported here, preliminary searches were performed directly on the MG-RAST server using key words “NEON” and “National Ecological Observatory Network”. The initial search results returned 1,304 samples hosted by MG-RAST. These were further refined by ensuring that both “Amplicon” with a “target_gene” of “16S” and “WGS” sequencing methods were readily available under “sequence_type” for direct comparison. Additionally, the following criteria had to match across each of the retrieved sequences: 1) “collection_date” was from the same day; 2) “biome”, “biome_id”, and “feature” matched for each sample; and 3) the samples were collected from the same “location”. A total of 97 samples met these criteria, including 25 WGS samples and 51 amplicon samples. We further narrowed these search results by selecting four WGS and four amplicon sequencing method samples with the greatest number of sequencing reads from each year of collection and identified the area that contained the greatest number of entries. The refined samples selected for this study and their relevant metadata, including MG-RAST ID, NEON Data Product ID, NCBI BioProject ID, sequencing method, collection date, and collection location, are detailed in **Table 1**.

**Table 1:** Whole genome and 16S amplicon metagenomic datasets examined in this study.

MG-RAST ID	NEON Data Product ID	NCBI BioProject ID	Sequencing Method	Collection Date (M/D/Y)	Collection Location
mgm4637825.3	NEON Soil Metagenomes (DP1.10107.001)	PRJNA406974	WGS	4/15/14	40°49'06.4"N 104°42'25.8"W
mgm4637821.3	NEON Soil Metagenomes (DP1.10107.001)	PRJNA406974	WGS	4/15/14	40°49'06.3"N 104°42'25.2"W
mgm4637831.3	NEON Soil Metagenomes (DP1.10107.001)	PRJNA406974	WGS	7/15/14	40°48'45.9"N 104°41'48.7"W
mgm4637826.3	NEON Soil Metagenomes (DP1.10107.001)	PRJNA406974	WGS	7/16/14	40°49'06.4"N 104°42'25.8"W
mgm4783766.3	NEON Soil Marker Gene Sequences (DP1.10108.001)	PRJNA393362	16S Amplicon	4/15/14	40°51'02.8"N 104°41'58.9"W
mgm4783759.3	NEON Soil Marker Gene Sequences (DP1.10108.001)	PRJNA393362	16S Amplicon	4/15/14	40°51'02.9"N 104°41'57.8"W
mgm4778732.3	NEON Soil Marker Gene Sequences (DP1.10108.001)	PRJNA393362	16S Amplicon	7/15/14	40°51'03.0"N 104°41'58.8"W
mgm4778744.3	NEON Soil Marker Gene Sequences (DP1.10108.001)	PRJNA393362	16S Amplicon	7/16/14	40°49'02.6"N 104°45'00.9"W

All whole genome and 16S amplicon metagenomic samples are a part of the NEON soil microbial metagenomic sequencing and NEON soil microbe marker gene sequencing projects (National Science Foundation, Grant #1638694, Grant ID MREFC), respectively, collected with the overall goal of tracking changes in the diversity, composition, and functional potential of microbiota in soil ecosystems through time and space. NEON collects surface soil samples to 30 cm depth. All samples were collected in a temperate grassland biome (biome_id = ENVO:01000193) with features of graminoid or herbaceous vegetation from Central Plains Experimental Range, Colorado, USA [44]. MG-RAST ID, NEON Data Product ID, NCBI BioProject ID, sequencing method, collection date, and location of the collection site are given. Metagenomic sequences were generated on the Illumina HiSeq and MiSeq instruments for WGS and 16S amplicon sequencing methods, respectively, and all samples are publicly available in the MG-RAST server and NCBI.

### Identification employing metagenomic sequencing reads

Sample collection, DNA preparation, and sequencing were performed by NEON investigators using standardized operating procedures. Briefly, NEON samples were collected from surface soil down to 30 cm in depth, frozen on dry ice, and shipped to a NEON analytical facility for DNA extraction, sample preparation, and sequencing on Illumina HiSeq and MiSeq instruments for WGS and 16S amplicon sequencing methods, respectively. **Table 2** details general analysis statistics, including the number of sequencing reads, mean sequence length, identified protein and rRNA features, and taxonomic hits distribution. Quality control (QC) thresholds were maintained internally through the MG-RAST automated processing pipeline, and no further QC cutoffs were applied to NEON sequences. The number of identified protein and rRNA features is a result of the contig lowest common ancestor (contigLCA) algorithm used by the MG-RAST automated pipeline to find a single consensus taxonomic entry for all features on each individual sequence, with default cutoffs for alignment length, e-value, and percent identity of the raw sequencing reads against the nonredundant M5NR database [46] that contains sequences and annotations from multiple publicly available sources to maintain two databases for protein and ribosomal sequence data. The MG-RAST annotation pipeline has the potential to map one read to multiple annotations and map one annotation to multiple reads. Therefore, “hits” are an estimate of the number of sequences that contain a given annotation, found by multiplying each database hit by the number of representatives in each cluster. Accordingly, “hits” refers to the number of unique database sequences that were found following a similarity search and not the number of reads. Therefore, the number of identified features can be smaller than the number of reads due to clustering or larger due to double counting. Protein database sources include GO, IMG, KEGG, NCBI (RefSeq and GenBank), SEED, UniProt, eggnog, and PATRIC and ribosomal database sources include RDP, SILVA, and Greengenes. Total taxonomic hits, and taxonomic hits for archaea, bacteria, eukaryota, and viruses, were determined using the contigLCA algorithm against the M5NR database for samples analyzed via WGS (MG-RAST metagenome identification numbers = mgm4637825.3, mgm4637821.3, mgm4637831.3, and mgm4637826.3). Analogously, total taxonomic hits, and taxonomic hits for archaea, bacteria, and eukaryota were determined for samples analyzed using 16S amplicon sequencing (MG-RAST metagenome identification numbers = mgm4783766.3, mgm4783759.3, mgm4778732.3, and mgm4778744.3).

**Table 2:** Sequence breakdown of quality predicted protein features, and total taxonomic hits of WGS and 16S amplicon sequencing samples included in this study.

							Taxonomic Hits Distribution (Relative Abundance %)				
MG-RAST ID	Sequencing Method	Sequence Count Post QC	Mean Sequence Length	Identified Protein Features	Identified rRNA Features	Total Taxonomic Hits	Archaea	Bacteria	Eukaryota	Viruses	Other Sequences
mgm4637825.3	WGS	11,623,197	158 ± 14 bp	3,637,507	4,365	3,349,527	27,103 (0.81%)	3,280,081 (97.93%)	36,533 (1.09%)	385 (0.01%)	5,425 (0.16%)
mgm4637821.3	WGS	11,088,780	162 ± 16 bp	3,575,354	3,603	3,285,741	24,348 (0.74%)	3,236,387 (98.50%)	19,219 (0.58%)	324 (0.01%)	5,463 (0.17%)
mgm4637831.3	WGS	5,704,956	162 ± 15 bp	1,748,119	2,264	1,621,138	11,851 (0.73%)	1,594,072 (98.33%)	12,309 (0.76%)	279 (0.02%)	2,627 (0.16%)
mgm4637826.3	WGS	5,663,984	159 ± 14 bp	1,823,419	2,588	1,679,821	11,619 (0.69%)	1,651,457 (98.31%)	14,006 (0.83%)	240 (0.01%)	2,499 (0.16%)
mgm4783766.3	16S	2,827	253 ± 2 bp	N/A	2,752	18,197	713 (5.57%)	10,548 (82.43%)	946 (7.39%)	0	5,990 (4.61%)
mgm4783759.3	16S	2,420	253 ± 2 bp	N/A	2,765	9,728	631 (6.49%)	8,262 (84.93%)	491 (5.05%)	0	644 (3.53%)
mgm4778732.3	16S	5,132	253 ± 2 bp	N/A	5,043	23,807	737 (3.10%)	21,054 (88.44%)	1309 (5.5%)	0	707 (2.96%)
mgm4778744.3	16S	2,880	253 ± 3 bp	N/A	3,643	10,860	393 (3.62%)	9,626 (88.64%)	657 (6.05%)	0	184 (1.69%)

WGS and 16S amplicon sequencing samples were sequenced on Illumina HiSeq and MiSeq sequencing platforms, respectively. Quality control and predicted protein features are a result of the automated analysis generated by the MG-RAST pipeline. Total taxonomic hits were determined using a contigLCA algorithm to find a single consensus taxonomic entry for all features on each individual sequence with the default cutoffs for alignment length, e-value, and percent identity against the M5NR database for NEON samples sequenced via WGS (mgm4637825.3, mgm4637821.3, mgm4637831.3, and mgm4637826.3) and 16S amplicon (mgm4783766.3, mgm4783759.3, mgm4778732.3, and mgm4778744.3) methods, respectively. Database hits refer to the number of unique database sequences that were found following a similarity search employed by the MG-RAST annotation pipeline and not the number of reads. Therefore, the number of identified features can be smaller than the number of reads due to clustering or larger due to double counting. MG-RAST ID, sequence count, mean sequence length, identified protein features, identified rRNA features, total taxonomic hits, and the taxonomic hits distribution of archaea, bacteria, eukaryota, viruses, and unclassified reads are given.

**Fig 1:**
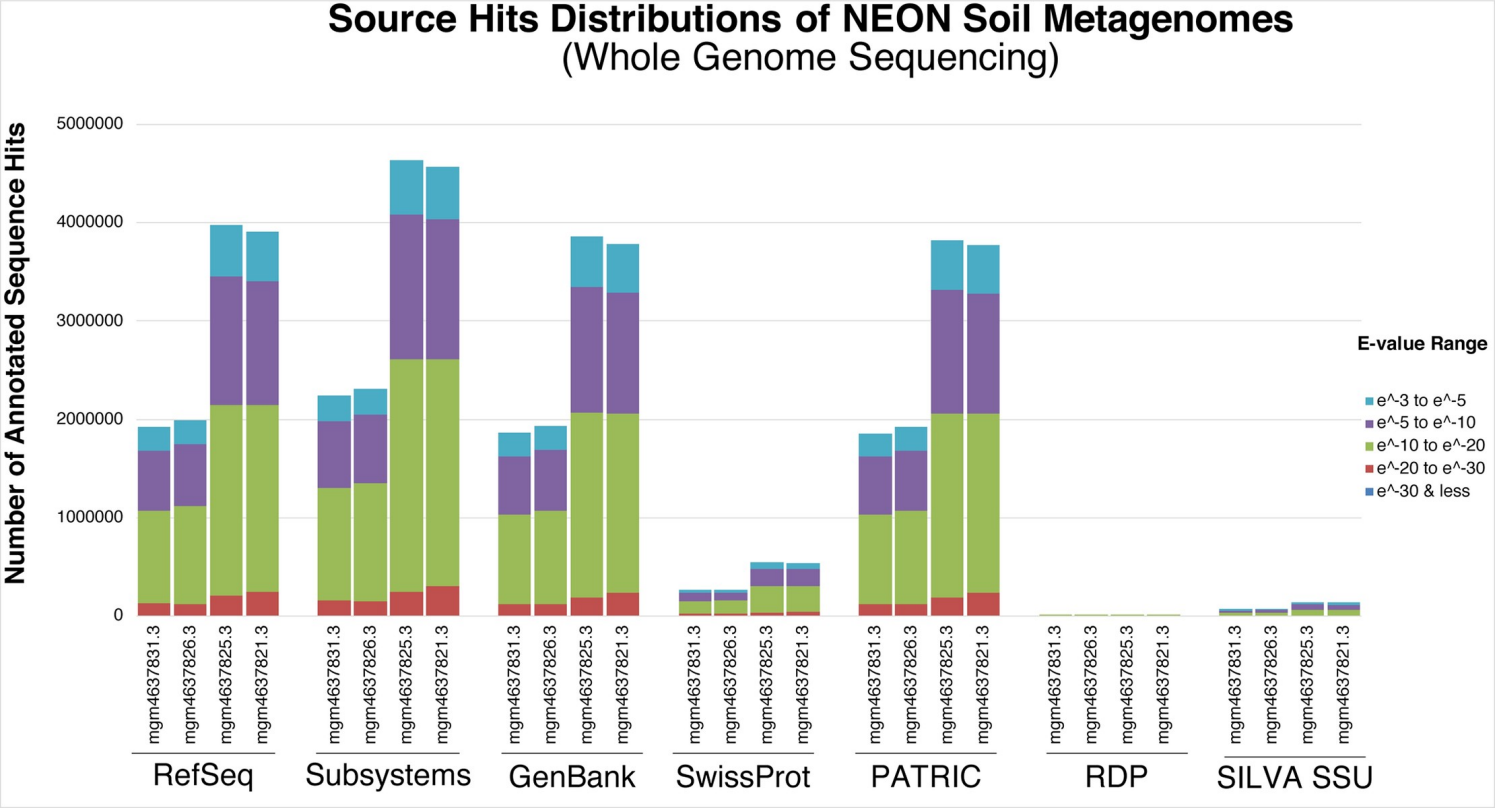
Source hits distribution of WGS metagenomic samples showing the number of annotated read hits across different databases. Databases include protein databases with functional hierarchy information, and ribosomal RNA databases. Colored bars represent annotated reads colored by the observed e-value range. Databases differ in the number of hits, but also have different types of annotation data.

**Fig 2:**
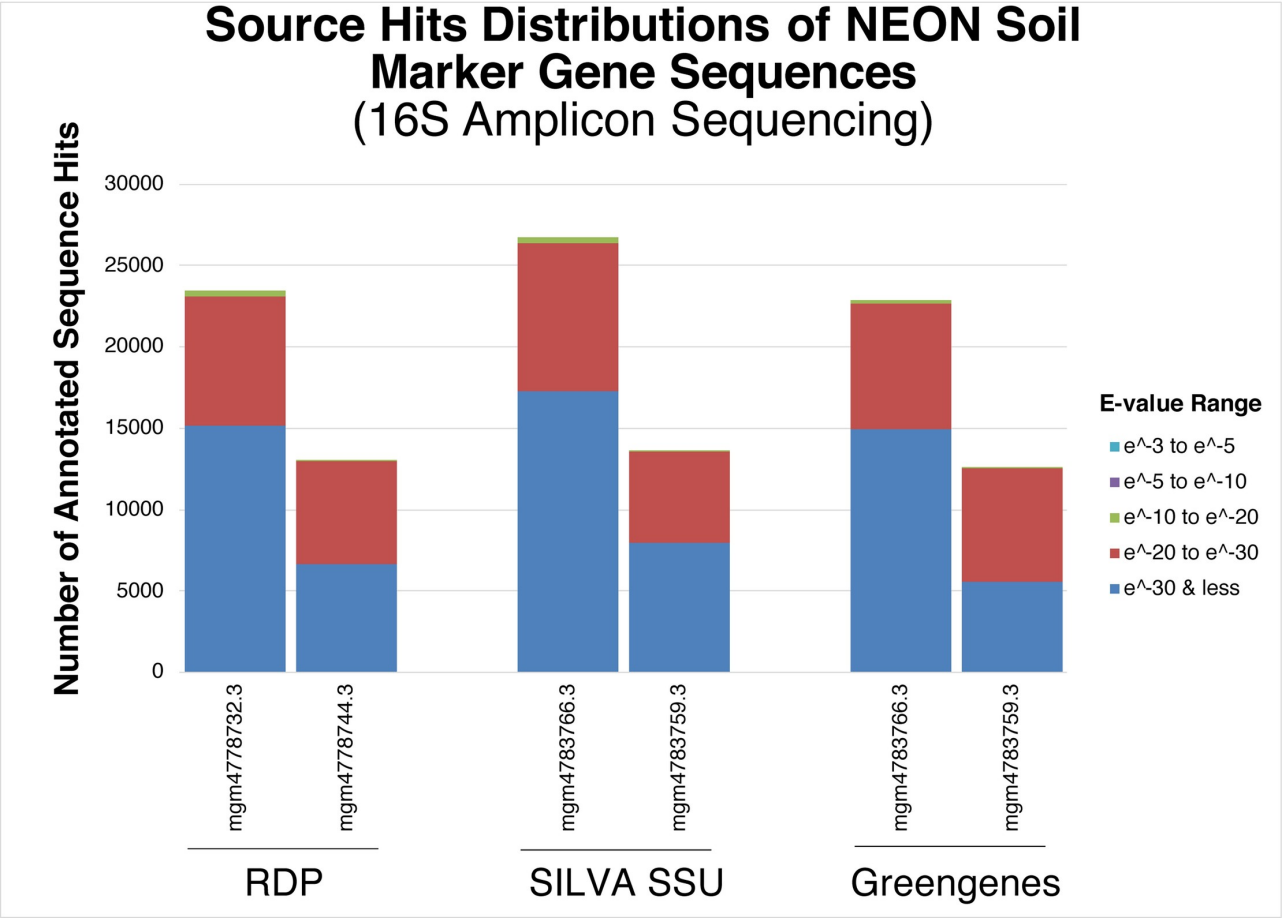
Source hits distribution of 16S amplicon sequencing metagenomic samples showing the number of annotated read hits across different databases. Shown are ribosomal RNA databases, RDP, SILVA SSU, and Greengenes. Colored bars represent annotated reads colored by the observed e-value range. Databases differ in the number of hits, but also have different types of annotation data. Source hit distribution information was not available for mgm4783766.3 and mgm4783759.3 at the time of analysis.

The contigLCA was also used to determine the individual source hits distributions of WGS NEON soil metagenomes (mgm4637825.3, mgm4637821.3, mgm4637831.3, and mgm4637826.3) against RefSeq, subsystems of SEED level-one functions, GenBank SwissProt, PATRIC, RDP, and SILVA SSU databases (**Fig 1**). The source hits distribution of 16S amplicon NEON Soil Marker Gene Sequences (mgm4778732.3 and mgm4778744.3) was determined using the contigLCA algorithm against RDP, SILVA SSU, and Greengenes databases (**Fig 2**). Data for the source hits distribution of mgm4789766.3 and mgm4783759.3 was not available on the MG-RAST server at the time of analysis.

**Fig 3:**
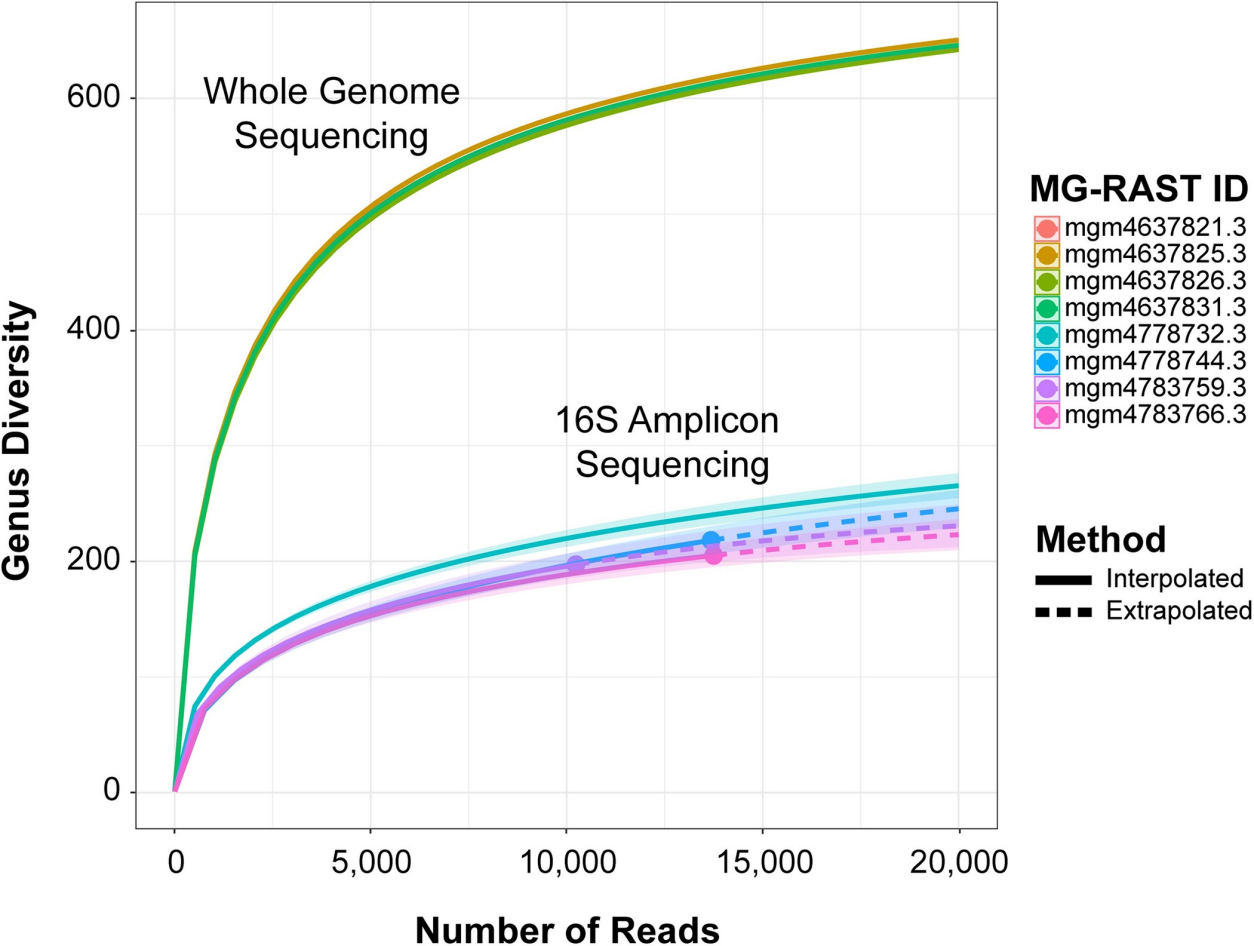
Genus richness estimates for rarefied and extrapolated samples of classified bacteria, archaea, eukaryota, and viruses from WGS show greater genus diversity compared to 16S amplicon sequencing, with respect to read number. Whole-genome sequencing and 16S amplicon sequencing methods are visualized as genetic diversity compared to number of sequencing reads for each metagenomic sample set mapped against RefSeq and RDP databases, respectively, using contig LCA algorithm on the MG-RAST server [45]. Shaded areas represent 95% confidence intervals with 1,000 bootstrap repetitions, interpolation (solid lines) and extrapolation (dotted lines) curves were generated using the R software package ‘iNEXT’ [47]. Rarefaction curves are separated by color for each sample. Solid line interpolated; dashed line, extrapolated.

The MG-RAST metagenomics analysis server provides rarefaction curves as the total number of distinct species annotations, a function of the number of sequencing reads. However, MG-RAST recommends against using shotgun sequence data to infer taxonomic information below genus for direct analysis. Therefore, to examine organism diversity, a rarefaction curve was created independently to examine genus richness (**Fig 3**). Briefly, unassembled metagenomic sequencing reads were first analyzed on the MG-RAST server using the contigLCA algorithm to map the raw sequencing reads directly to the RefSeq and RDP databases for the WGS and 16S amplicon sequencing samples, respectively. The corresponding read abundance values were used to create sample-size-based rarefaction (interpolation) and extrapolation (prediction) curves with an endpoint of 20,000 reads and 1,000 bootstrap repetitions using the R software package ‘iNEXT’ [47]. Hill numbers, i.e., the effective number of genera, were used to quantify the taxonomic diversity of each assemblage, that is, the sampling curve plots diversity estimates with respect to the number of sampling units, i.e., the number of reads [48–50]. The curve represents the estimated number of different genus annotations for subsamples of each complete dataset and predicts accurately up to double the reference sample size [47]. Therefore, as the curve becomes flatter towards the distal part of the x-axis, additional sampling is likely to yield fewer new genera identifications. Sample-size-based rarefaction curves extrapolated to twice the sample size of WGS samples examined in this study are provided in the supporting information (**S1 Fig**)

**Fig 4:**
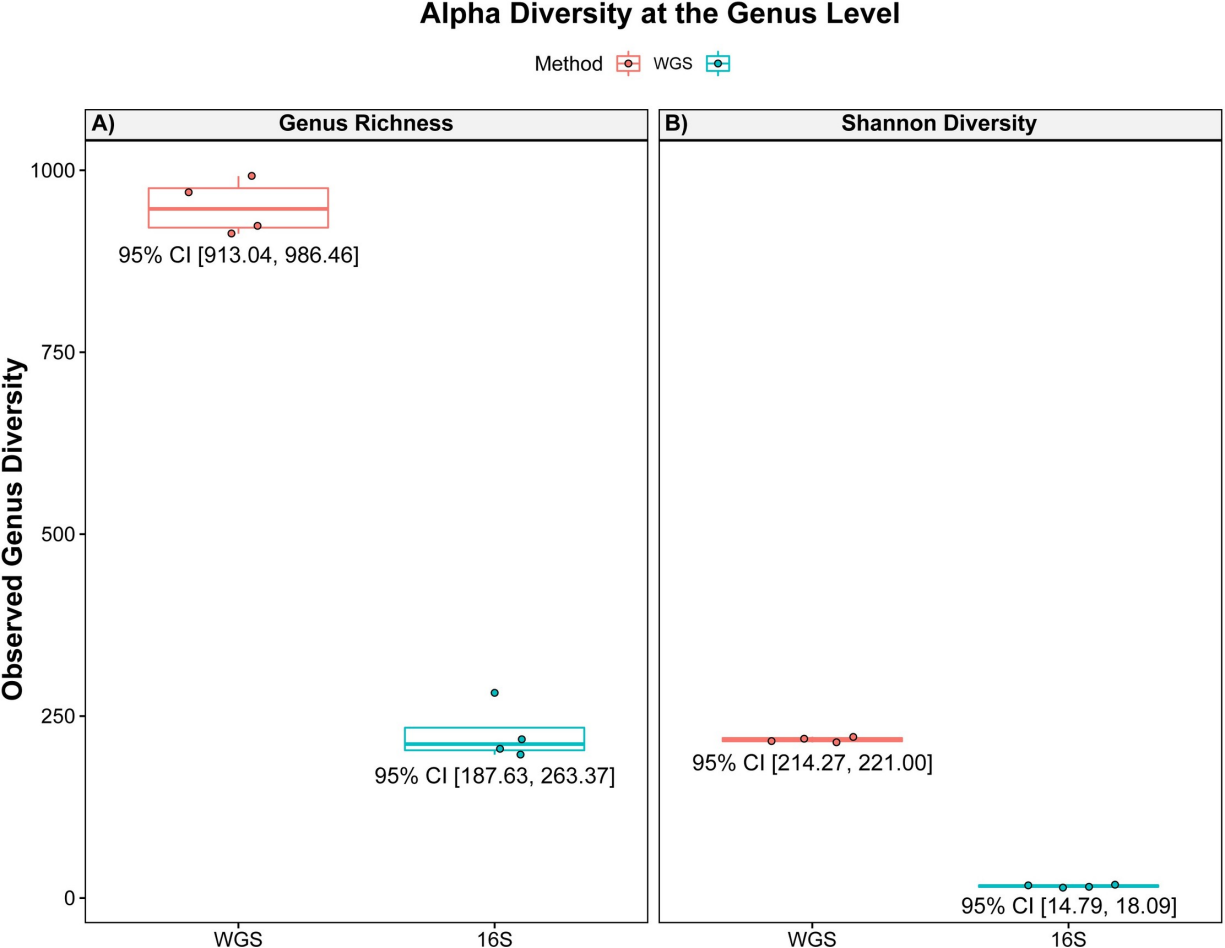
Boxplots of the alpha diversity observed at the genus level. (A) the observed number of annotated genera and (B) Shannon index alpha-diversity values of WGS (mgm4637825.3; mgm4637821.3; mgm4637831.3; mgm4637826.3) and 16S amplicon sequencing (mgm4783766.3; mgm4783759.3; mgm4778732.3; mgm4778744.3) samples included in this study. Shannon’s index alpha diversity was calculated using the R software package ‘iNEXT’ [47]. The 95 percent confidence intervals (95% CI) were calculated using the Z statistic for WGS and 16S amplicon sequencing indicated for each sample set. Y-axis is shared between panels. Boxplots are separated by color for WGS and 16S samples.

Boxplots were used to summarize alpha diversity of annotated genera in each sample (**Fig 4**). Alpha diversity is shown as the total number of annotated genera in each sample (**Fig 4A**) and the genus richness calculated using Shannon’s index (**Fig 4B**) from the corresponding read abundance values matrix obtained from MG-RAST for WGS and 16S amplicon sequencing samples, as previously mentioned. Shannon’s index alpha diversity was calculated using the R software package ‘iNEXT’ [47], and 95 percent confidence intervals (95% CI) were calculated using the Z statistic for WGS and 16S amplicon sequencing.

**Fig 5:**
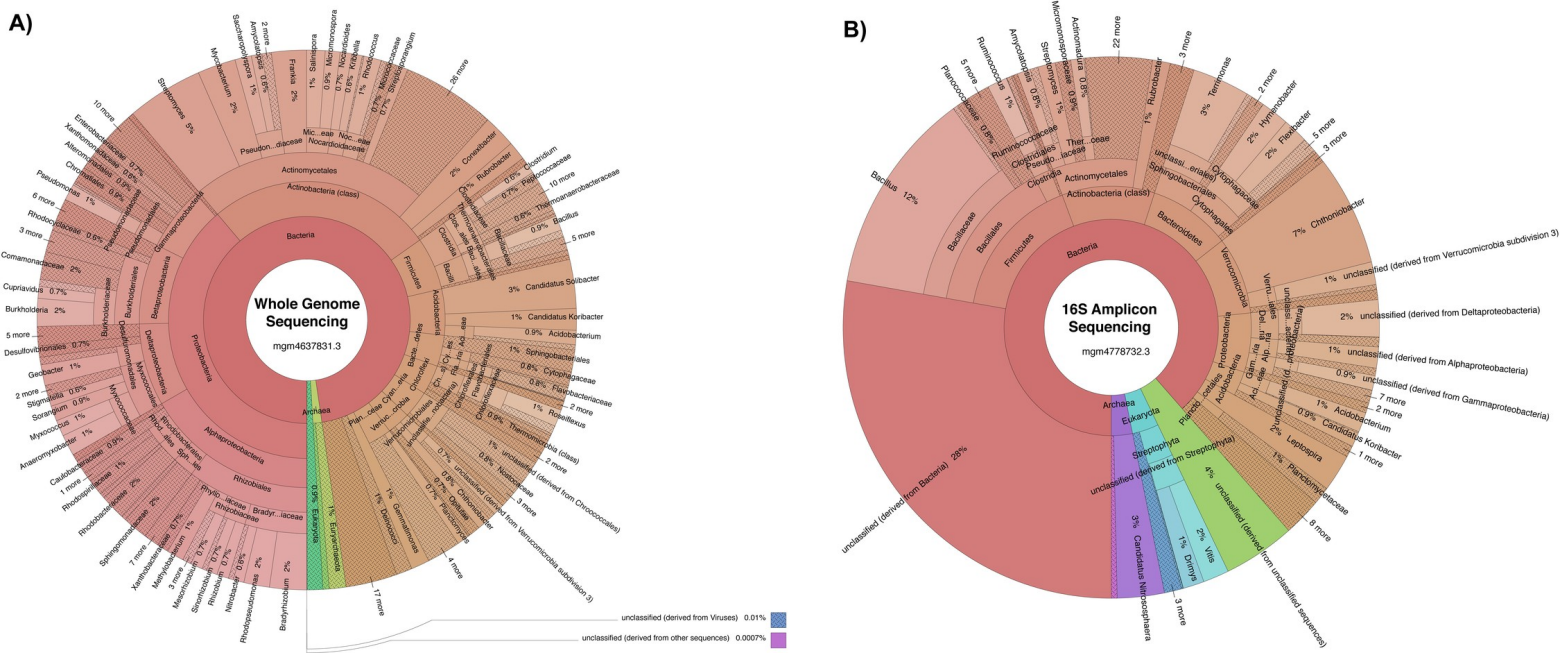
Krona plots of the microbiome detected in representative metagenomic datasets. (A) Whole-genome sequencing sample mgm4637831.3 and (B) 16S amplicon sequencing sample mgm4778732.3 are visualized as genetic diversity identified at the genus level. Taxonomic nodes are nested sectors arranged from the top level of the hierarchy at the center and progressing outward. Krona plots simultaneously display relative abundance and hierarchy using a radial space-filling display. Genera composition percentages are displayed as the normalized proportion of organism specific k-mers observed in representative datasets. Taxonomic domains are separated by color for bacteria, eukaryota, archaea, viruses, and unclassified reads, respectively.

**Fig 6:**
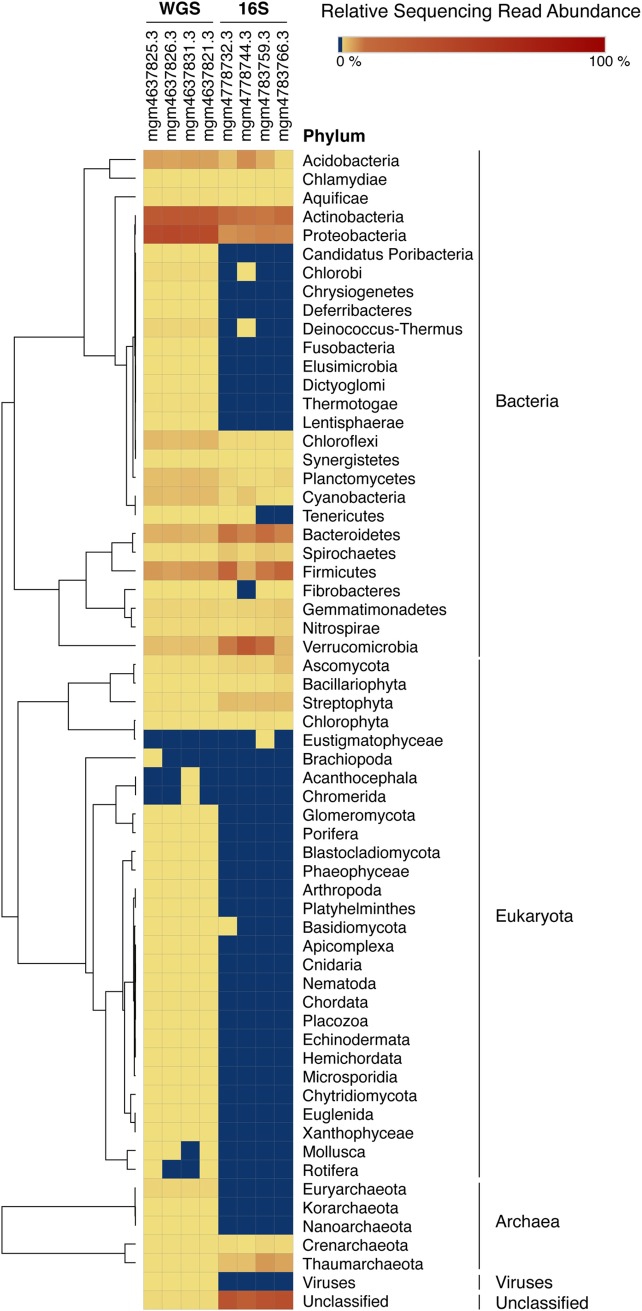
Heatmap of relative abundance of microbial phyla detected in WGS and 16S amplicon sequencing samples. Composition percentages are displayed as the normalized proportion of the phylum specific k-mers observed in each sample relative to the total microbial phyla diversity of the sample. Color gradient key displays the scale of relative abundance percentages for WGS (mgm4637825.3, mgm4637821.3, mgm4637831.3, and mgm4637826.3) and 16S amplicon sequencing (mgm4783766.3, mgm4783759.3, mgm4778732.3, and mgm4778744.3). Hierarchical row clustering was generated using one minus Pearson correlation.

Certain bioinformatics software packages, for example, CosmosID, Inc. (CosmosID, Inc., Rockville, MD, USA), Kraken2 [51,52], MetaPhlAn [53], and MetaMaps [54], are able to achieve bacterial identification to species, subspecies, and/or strain level using unassembled metagenomic shotgun [55–58] and long sequencing reads [54]. MG-RAST metagenomics analysis server recommends against using shotgun sequence data to infer taxonomic information below the genus level for direct analysis. Accordingly, using the contigLCA algorithm on the MG-RAST server, genus-level taxonomic categories from each metagenome were determined by mapping the raw sequencing reads directly to the RefSeq and RDP databases for WGS and 16S amplicon sequencing, respectively. Sunburst visualizations of organism specific k-mer relative abundance (percentage) for each sample were generated using Krona [59] (**Fig 5** **and S2–S7 Figs**). To compare the overall predicted relative abundance of bacteria, eukaryota, archaea, viruses, and total unclassified sequences detected in each sample, a heatmap of phylum specific k-mer relative abundance (percentage) observed in each sample was created using Morpheus with hierarchical row clustering and One Minus Pearson Correlation [60] (**Fig 6**). Fungal internal transcribed spacer (ITS) genomic marker regions were not evaluated in this study.

## Results

### NEON samples identified for metagenomic sequencing analyses

A total of four whole genome shotgun (mgm4637825.3, mgm4637821.3, mgm4637831.3, mgm4637826.3) and four 16S amplicon (mgm4783766.3, mgm4783759.3, mgm4778732.3, mgm4778744.3) metagenomic samples were selected after performing searches on the MG-RAST server with predefined filtering criteria and identifying samples with the greatest number of sequencing reads collected from the same location. Prior to sequencing, all samples had been collected by NEON researchers from surface soil at 30 cm in depth in a temperate grassland biome (biome_id = ENVO:01000193) with features of graminoid or herbaceous vegetation from Central Plains Experimental Range, Colorado, USA between April-July 2014 (**Table 1**).

### Taxonomic hits distribution against MG-RAST M5NR database

The number of predicted protein features, identified rRNA features, and total taxonomic hits resulting from the automated analysis generated by the MG-RAST pipeline by mapping raw sequencing reads against the M5NR database using the contigLCA algorithm are detailed in **Table 2**. Samples analyzed by the WGS method employing Illumina HiSeq chemistry produced between 5,66,108 and 11,627,943 (average = 8,525,007) sequencing reads with mean sequence read lengths between 144 bp and 177 bp, compared to the 16S amplicon sequencing method employing Illumina MiSeq chemistry, which produced between 15,799 and 37,106 (average = 22,886) sequencing reads with mean sequence read lengths of between 250 bp and 256 bp. Raw sequencing reads were mapped against the M5NR database and the 16S amplicon sequencing contained, on average, a greater number of identified rRNA features (average = 3,550.75) compared to the WGS sequencing method (average = 3,205). Additionally, the number of total taxonomic hits of raw sequencing reads against the M5NR database was greater for WGS, ranging between 1,621,138 and 3,349,527 (average = 2,484,057), compared to total taxonomic hits of the 16S amplicon sequencing which ranged between 9,728 and 23,807 (average = 15,648). Across all samples, relative abundance of taxonomic hits was greater for archaea and eukaryota in 16S amplicon sequencing (average = 4.70% and 6.00%, respectively) compared to the WGS sequencing method (average = 0.74% and 0.81%, respectively). Conversely, relative abundance of bacteria was slightly decreased in 16S amplicon sequencing (average = 86.11%) compared to the WGS method (average = 98.27%). No viruses were detected in the 16S amplicon sequencing samples while between 0.01% and 0.02% of the reads mapped to viruses in the shotgun metagenomic samples. 16S amplicon sequencing targets DNA sequences encoding the RNA component of the 30S subunit of prokaryotic ribosomes and is not useful in identifying functional protein features, while between 1,823,429 and 3,637,507 (average = 2,696,100) protein features were identified in the WGS samples. Additionally, the proportion of ‘other and unclassified’ sequencing reads was greater in the 16S amplicon sequencing samples (average = 3.20%) compared to the shotgun metagenomic sequencing samples (average = 0.16%).

### Source hits distribution against other widely used databases

The source hits distribution was determined by using the contigLCA algorithm to map raw sequencing reads against individual databases for the WGS sequencing method (**Fig 1**) and the 16S amplicon sequencing (**Fig 2**). The average total number of raw WGS reads mapped against whole genome taxonomic databases, RefSeq, GenBank, and PATRIC, was 2,950,350.75, 2,860,207, and 2,842,863.5, respectively. The average total of raw WGS reads mapped against genome annotations and protein databases, Subsystems ontology and SwissProt, was 2,860,207 and 407,185, respectively. All WGS samples, and two 16S amplicon samples (mgm4778732.3 and mgm4778744.3) were mapped against rRNA databases RDP and Silva SSU. The 16S amplicon sequencing samples demonstrated a greater number of hits against the RDP database, 23,442 and 13,072 hits for mgm4778732.3 and mgm4778744.3, respectively (average = 18,257) compared to the number of WGS sample reads mapped against the RDP database, which ranged between 1,563 and 3,505 (average = 2,416.5). However, WGS metagenomic samples contained a larger number of hits against the Silva SSU database, which ranged between 69,560 and 142,953 (average = 106180.25), compared to the 16S amplicon sequencing methods, which demonstrated 13,662 and 22,876 hits for mgm4778732.3 and mgm4778744.3, respectively (average = 20,188). The 16S amplicon sequencing samples mapped against the Greengenes database showed 22,876 and 12,633 hits for mgm4778732.3 and mgm4778744.3, respectively (average = 20,188). By default, the MG-RAST analysis server does not provide the source hits distribution of WGS reads mapped against the Greengenes database. Therefore, no comparison was made between WGS samples and the Greengenes database.

### Rarefaction and Shannon’s index alpha diversity

Overall genus richness, i.e., the total number of phylotypes, of each sample microbiome sequenced by WGS and 16S amplicon sequencing was compared by construction of rarefaction curves (**Fig 3**). Across all samples, genera richness in WGS samples was consistently greater than for samples sequenced by the 16S amplicon sequencing method. Furthermore, rarefaction analysis indicated that coverage was sufficient using 16S amplicon sequencing samples, as the asymptote of the curve towards the end of the graph is nearly zero, whereas, for the WGS shotgun metagenomic samples, increasing coverage would likely result in an increased number of samples being identified, as the asymptote of the curve had not reached maximum genus richness, supported by the extrapolated rarefaction sampling curves of WGS samples (**S1 Fig**). The total number of annotated genera observed in each sample is shown in in **Fig 4A**. The number of annotated genera observed in WGS samples (mgm4637825.3, 991; mgm4637821.3, 969; mgm4637831.3, 923; mgm4637826.3, 912; average = 948.75, 95% CI [913.04, 986.46]) was significantly greater, compared to 16S amplicon sequencing samples (mgm4783766.3, 204; mgm4783759.3, 196; mgm4778732.3, 281; mgm4778744.3, 217; average = 224.5; 95% CI = [187.63, 263.37]) selected in this study.

Similarly, alpha diversity patterns were calculated at the genus level using Shannon’s index for NEON soil metagenomes analyzed via WGS andNEON Soil Marker Gene Sequences analyzed via 16S amplicon sequencing selected in this study (**Fig 4B**). The observed Shannon index alpha diversity values were greater for WGS samples (mgm4637825.3, 221.56; mgm4637821.3, 219.93; mgm4637831.3, 215.74; mgm4637826.3, 213.94) compared to 16S samples (mgm4783766.3, 15.57; mgm4783759.3, 17.21; mgm4778732.3, 18.37; mgm4778744.3, 14.59) selected in this study. Across NEON soil metagenomes obtained by WGS, Shannon index alpha diversity was significantly greater (average = 217.79, 95% CI = [214.27, 221.00]) than alpha diversity of all NEON Soil Marker Gene Sequences (average = 16.44; 95% CI = [14.79, 18.09]) obtained by 16S amplicon sequencing.

### Microbial resolution of WGS and 16S amplicon sequencing

The average total number of taxonomic hits for WGS samples against the RefSeq database was 4,780,201.75 (mgm4637825.3 = 6,445,937; mgm4637821.3 = 6,347,609; mgm4637831.3 = 3,102,947; mgm4637826.3 = 3,224,314). The average total number of taxonomic hits for 16S amplicon sequencing against the RDP database was 15,785.75 (mgm4783766.3 = 13,751; mgm4783759.3 = 10,237; mgm4778732.3 = 25,470; mgm4778744.3 = 13,685). To display and compare information for the most abundant taxa across samples, characterization of DNA coding for bacteria, archaea, eukaryota, and viruses are shown as Krona plots representing the relative abundance of microbial genera detected in the representative WGS sample mgm4637831.3 (**Fig 5A**) and 16S amplicon sequencing representative sample mgm4778732.3 (**Fig 5B**). Krona plots for other WGS samples (mgm4637825.3, mgm4637821.3, and mgm4637826.3) and 16S amplicon sequencing samples (mgm4783766.3, mgm4783759.3, and mgm4778744.3) are provided in supporting information (**S2, S3 and S4 Figs**) and (**S5, S6 and S7 Figs**), respectively. Samples analyzed by WGS had a much lower proportion of unclassified reads compared to samples analyzed by 16S amplicon sequencing. For example, mgm4637831.3 (WGS) contained 67,939 reads unclassified to genus corresponding to approximately 2.19% of the total number of processed reads. In mgm4637831.3, approximately 923 genera were identified. In the 16S amplicon sequencing sample mgm4778732.3, 10,119 reads were unclassified at the genus level, corresponding to approximately 39.73% of the total number of processed reads. A total of 281 genera were identified in mgm4778732.3.

To compare dominant taxonomic groups detected by each sequencing method, relative abundance of microbial phyla detected in all WGS and 16S amplicon sequencing samples are shown in a heat map (**Fig 6**). Overall, dominant phyla detected in all samples agreed across sequencing methodology. With the exception of *Eustigmatophyceae* that was identified in mgm4783759.3, all phyla detected by 16S sequencing were also detected using WGS. Predominant bacterial phyla detected by both sequencing methods in all samples include *Acidobacteria*, *Actinobacteria*, *Proteobacteria*, *Bacteroidetes*, *Firmicutes*, and *Verrucomicrobia*. For all samples, WGS yielded more phyla compared to 16S amplicon sequencing. The detected diversity of eukaryota was greater across all samples using WGS compared to 16S, for which only *Basidiomycota* was detected. Similarly, five archaeal phyla were detected using WGS, while only *Crenarchaeota* and *Thaumarchaeota* were detected by 16S amplicon sequencing. WGS was also able to detect viruses at up to roughly 0.2% relative abundance in each sample examined, while no viruses were detected by 16S amplicon sequencing.

## Discussion

### WGS metagenomics offers finer resolution for microbial community structure and dynamics compared to 16S amplicon sequencing

Two most commonly employed methods of sequencing used to study the microbiome of complex environments are 16S rRNA sequencing and whole genome shotgun metagenomics. While, it is debatable as to which approach is superior, there may be a place for both in microbiome studies, depending on the investigation. Tessler and colleagues found that 16S sequencing identified a larger number of phyla than WGS for water samples collected across remote locations of Brazil, suggesting amplicon sequencing may outperform WGS in areas not well studied and comprising only a limited number of sequenced genomes [61]. WGS was concluded to be preferable to 16S amplicon sequencing in the human microbiome, including enhanced detection of bacterial species, increased detection of diversity, and increased prediction of genes [62]. Our findings support the latter, namely that WGS provides greater resolution, i.e., identifies greater microorganism diversity, and for microbial communities may provide greater insight into biochemical processes.

In general, amplicon sequencing may be more practical and less expensive than WGS, which may require more extensive data analysis [63–65]. Here we demonstrate that WGS offers insight into the total microbial community, and 16S amplicon sequencing identifies only more dominant organisms in a biological sample (**Fig 6**). Both amplicon sequencing and 16S targeting multiple loci, are useful in exploring biodiversity that includes bacterial, archaeal, and eukaryotic microbial communities in the same sequencing run [66]. A recent study demonstrated that MinION^TM^ technology can be employed to identify and differentiate both bacterial and viral species within a biological sample via amplicon sequencing [67]. However, WGS covers the entire community of genomes, capturing sequences of all organisms, including viruses and fungi, which cannot be captured by 16S amplicon sequencing. Additionally, 16S and WGS methods usually require different databases for classification of taxa [62].

In mining sequencing data from MG-RAST, we were unable to identify viral sequences using 16S amplicon sequencing, while whole genome metagenomic sequencing showed definitively that viral DNA comprised between 0.1%-0.2% of the total sequencing reads (**Table 2 and Fig 6**). Furthermore, 16S amplicon sequencing identified only very specific regions of the genome, insufficient to assess the functional genomics of microbial communities (**Figs 1 and 2**). Phylogenetic reconstruction has been employed by some investigators to infer biological function encoded in a genome containing a particular 16S sequence [68]. However, accuracy of such inferences relies heavily on how well the genomic diversity is represented by genomes available in the database [69]. Moreover, yet-to-be-discovered taxa whose rRNA sequences are not represented in the database would not be detected [70]. In this study, comparing 16S amplicon sequencing and WGS, we showed WGS metagenomics can be used effectively to identify predicted protein features whereas 16S amplicon sequencing could not reliably detect protein features (**Table 2**).

Identification of novel and highly complex organisms is difficult using 16S amplicon sequencing because the method is restricted to identifying those organisms whose specific genomic regions can be readily amplified. Furthermore, horizontal transfer of the 16S locus between distantly related taxa is possible [21]. Others have reported an overestimation of population diversity using 16S amplicon sequencing because many organisms in the environment contain multiple rRNA operons [21,71]. In this study, a larger proportion of taxonomic hits for archaea and eukaryota was obtained using 16S amplicon sequencing compared to WGS. Conversely, the distribution of hits for bacteria was slightly lower with 16S amplicon sequencing compared to WGS (**Table 2 and Fig 6**).

WGS shotgun metagenomics offers a deeper analysis of microbial diversity (**Figs 3 and 4A**). With rarefaction curves (**Figs 3 and S1**), we were able to demonstrate increased resolution of the taxonomic classification of microbial genera was not simply a result of number of reads generated in each sample, as shown by flattened curves in the rarefaction of 16S amplicon sequencing. Comparison of 16S amplicon sequencing with WGS metagenomic sequencing by rarefying reads from depths of 500 to 100,000 repeatedly, determined 16S amplicon sequencing can yield significant primer bias [72].

Shannon alpha genus diversity was found to be significantly greater for WGS samples compared to 16S amplicon metagenomic samples (**Fig 4B**), a finding in agreement with previous studies [62,73]. WGS also yielded enhanced detection of microbial diversity and accuracy. In the representative datasets, WGS identified 923 genera in sample mgm4637831.3 (**Fig 5A**), compared to 281 genera by 16S amplicon sequencing of sample mgm4778732.3 (**Fig 5B**).

With respect to cost, it was recently demonstrated that shallow WGS metagenomics can be used to obtain species-level taxonomic and functional data at a fraction of the cost of deep WGS and may serve as an alternative to 16S amplicon sequencing for large-scale microbiome studies [73].

### NEON data are promising for collaborative metagenomics and open source datamining

A major challenge of collaboration amongst researchers in metagenomics using existing open-source data for broad-scale analyses is the myriad of sequence databases that are available [74]. In these databases, metagenomics data can be stored in a variety of formats on distinct hardware and software platforms that are often isolated and independent from each other, with no standards established for data collection and communication. Each database is likely to require unique approaches and algorithms for data analysis, which can introduce additional variable interoperability [75]. Therefore, widely adopted standards would help investigators better utilize, share, and archive the ever-expanding volume of metagenomic data [76].

The NEON open source data portal is distinct because it has established a standard for data collection at sites in terrestrial and aquatic ecosystems that employ technical working groups to design protocols for data collection infrastructure, including sensor installation and configuration and supporting measurements, and for observations from samples collected at field sites [77]. NEON closely monitors all aspects of metagenomic analysis, including sample collection procedures, DNA preparation, and communication of observations and results. Variation of DNA preparation procedures have been shown to influence taxonomical classification using downstream sequencing reads [78,79] and can likely alter the identified microbial diversity profile. Currently, there is no uniform standard for ensuring complex metagenomic datasets are accompanied with relevant metadata across projects. However, NEON overcomes this by standardizing the protocols of sample collection and processing. In this study, we successfully demonstrated use of NEON metagenomic datasets (**Table 1**) with MG-RAST, an open-source standard for data analysis that provides support for automated phylogenetic and functional analysis of metagenome data [45]. NEON and MG-RAST collectively provide a means of standardized metagenomic data collection, processing, storage, analysis, and quality assurance which could be implemented in an array biodiversity studies related to use of long-term ecological data on a continental-scale.

### Limitations of applying standardized open source data

While implementation of the use of standardized open-source data is very appealing, it is not without limitation. The MG-RAST analytical server provides a standardized and reproducible platform for metagenomic analysis, but the documentation recommends against taxonomic classification below genus. Further, the MG-RAST annotation pipeline has potential to provide annotations for each submitted fragment of DNA such that the number of identified features may be smaller than the number of reads due to clustering or larger due to double counting. For many studies, species, and even strain level of taxonomic resolution can be essential to uncover fully all organisms present, including pathogenic strains, as well as identify dominant gene pathways that may be present in a sample. Accordingly, other curated databases (e.g., GenBook^®^) and analytical software platforms with strain resolution can be accessed and is appealing for studies where the goal is to identify microbial diversity and richness and to assign taxonomic or functional hierarchies.

Another limitation of using available open-source data is that investigators are limited to studying only the sequencing data that is readily available, therefore, designing projects that revolve around datasets that are available for a limited number of representative sites that may or may not provide the extent of diversity required for comparative metagenomics. In the case of NEON, data are released as available for regional to continental scale data collected and archived from 81 field sites across 20 ecoclimatic domains covering the contiguous 48 US states, Alaska, Hawaii, and Puerto Rico, and [80]. NEON provides a seamless integration with MG-RAST, which provides public access to calibrated meta and genomic data using standardized methods. As a result, the datasets can be used to formulate sampling sites, determine sampling frequency, and compare the metagenomic diversity and richness between samples.

## Conclusions

The National Ecological Observatory Network (NEON) provides regional to continental scale data gathered using standardized protocols and methods for sample collection, pre-processing, post-processing, and quality control. These data can be easily coupled with other standardized bioinformatic software (e.g., MG-RAST) for metagenomic analysis with a reproducible interoperability of results. In this study, we demonstrated the feasibility of using NEON metagenomic datasets to establish the resolution of microbial community structure and diversity. 16S amplicon sequencing is currently used to identify dominant organisms present in a biological sample. However, WGS has been shown to detect and identify more genera of bacteria, archaea, viruses, and eukaryota compared to 16S amplicon sequencing. Furthermore, the identification of putative functional genes in microbial communities provided significantly more effective using WGS than 16S amplicon sequencing. It is concluded that NEON open data are useful for characterizing and quantifying complex ecological processes associated with changing aquatic and terrestrial ecosystems. Other analytical software, in addition to MG-RAST, may be required to resolve taxonomic decisions below genus, i.e., species, strain, and sub-strain.

## Supporting information

S1 Fig Rarefaction curve of whole genome sequencing samples examined in this study. (TIF)

S2 Fig Krona plot of the microbiome detected in mgm4637826.3 whole genome sequencing dataset. (TIF)

S3 Fig Krona plot of the microbiome detected in mgm4637825.3 whole genome sequencing dataset. (TIF)

S4 Fig Krona plot of the microbiome detected inmgm4637821.3 whole genome sequencing dataset. (TIF)

S5 Fig Krona plot of the microbiome detected in mgm4778744.3 16S amplicon sequencing dataset. (TIF)

S6 Fig Krona plot of the microbiome detected in mgm4783759.3 16S amplicon sequencing dataset. (TIF)

S7 Fig Krona plot of the microbiome detected in mgm4783766.3 16S amplicon sequencing dataset. (TIF)
