# Supplementary figures and images for: Microbial resolution of whole genome shotgun and 16S amplicon metagenomic sequencing using publicly available NEON data
Source: PLoS One. 2020 Feb 13;15(2):e0228899. doi: 10.1371/journal.pone.0228899 (PMC7018008; doi:10.1371/journal.pone.0228899)

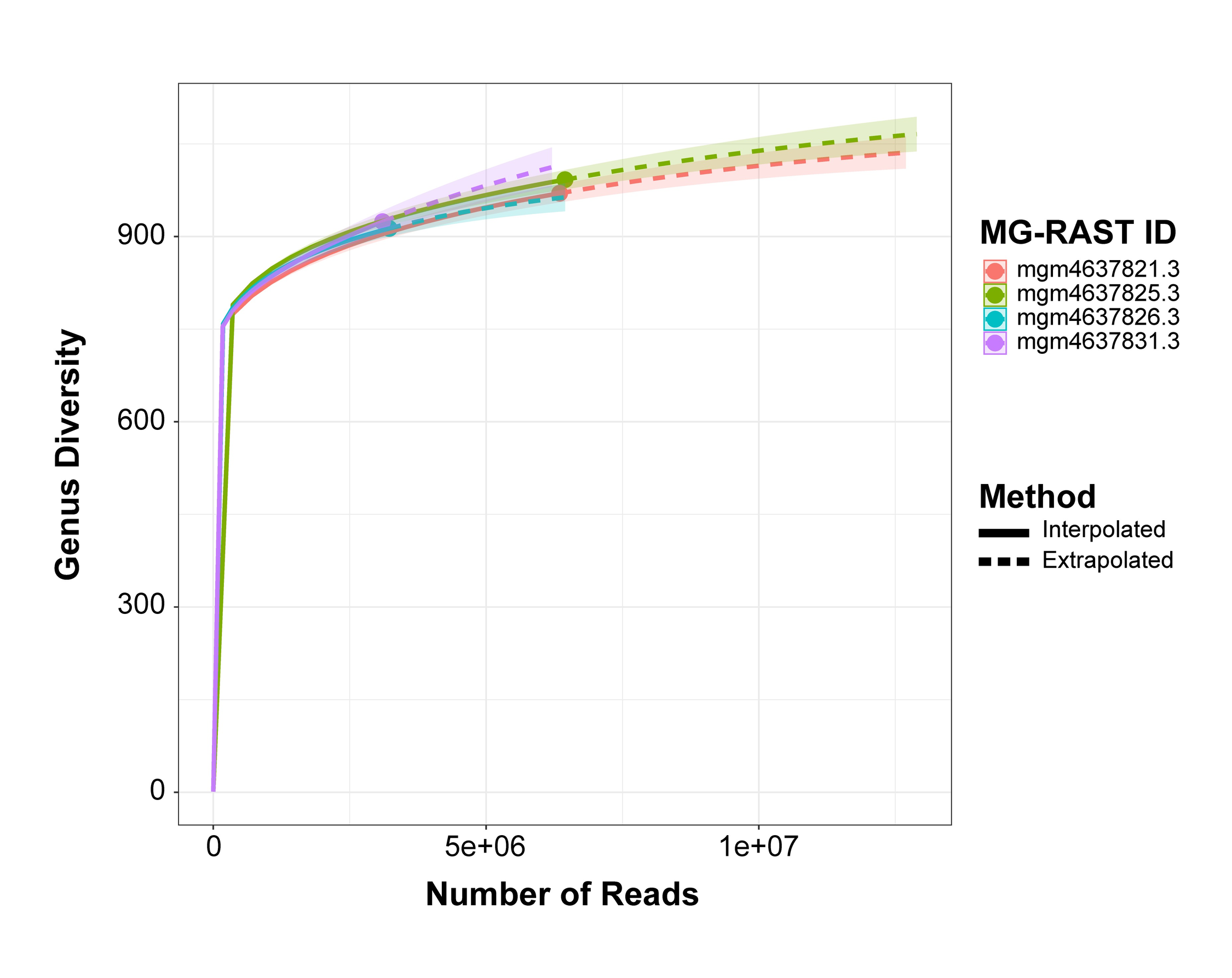

Supplement: S1 Fig — (TIF) [file pone.0228899.s001.tif]

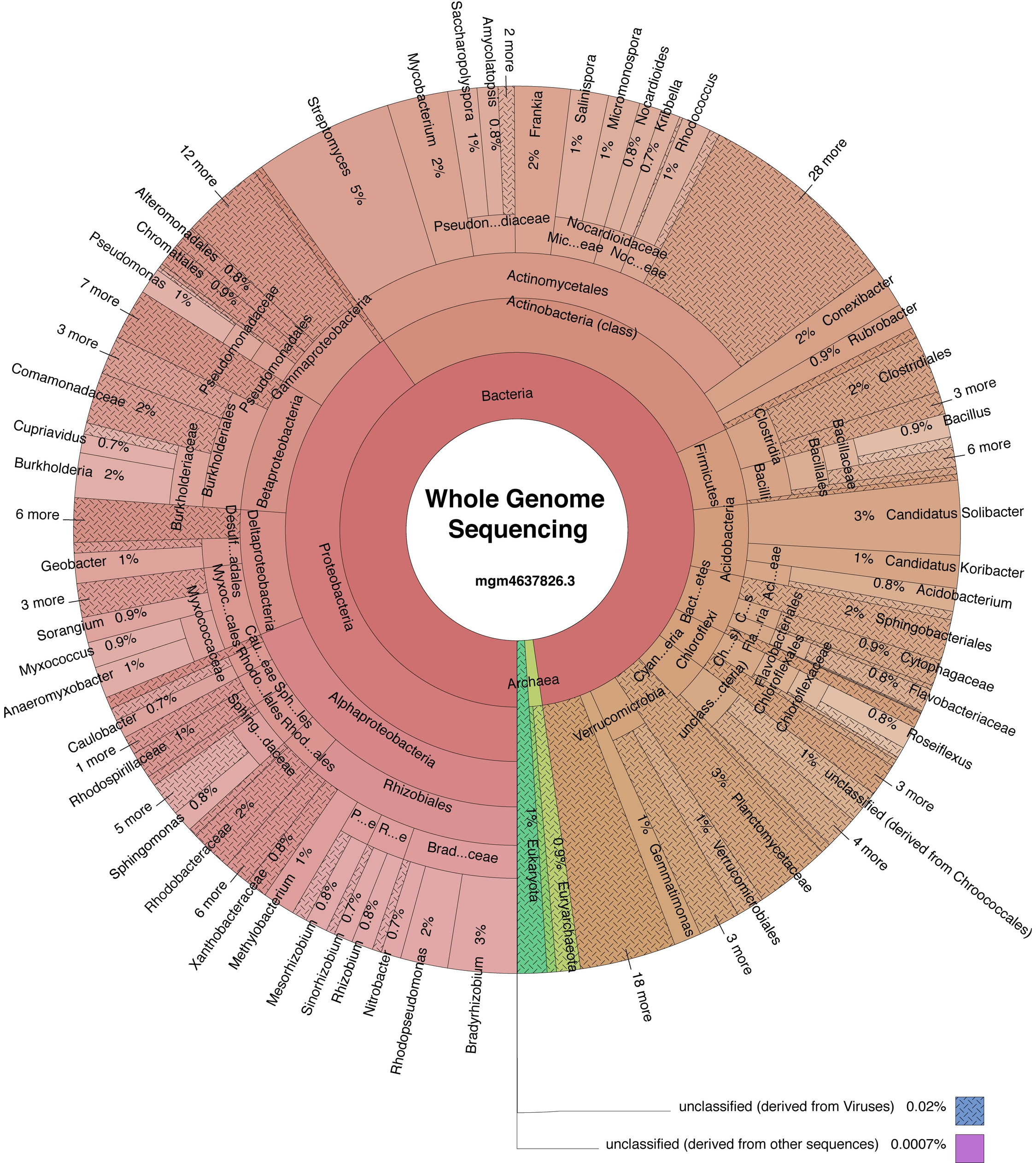

Supplement: S2 Fig — (TIF) [file pone.0228899.s002.tif]

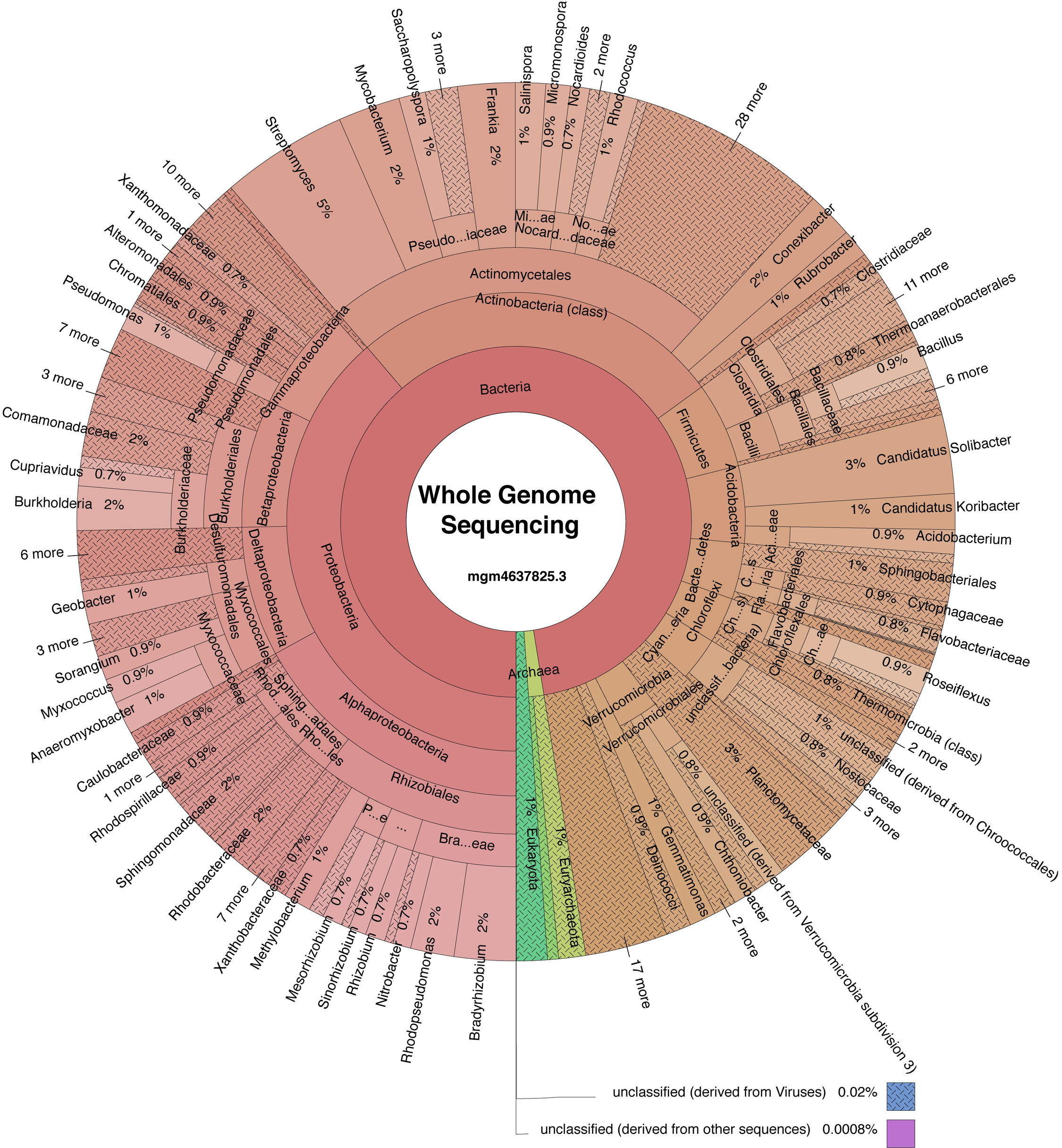

Supplement: S3 Fig — (TIF) [file pone.0228899.s003.tif]

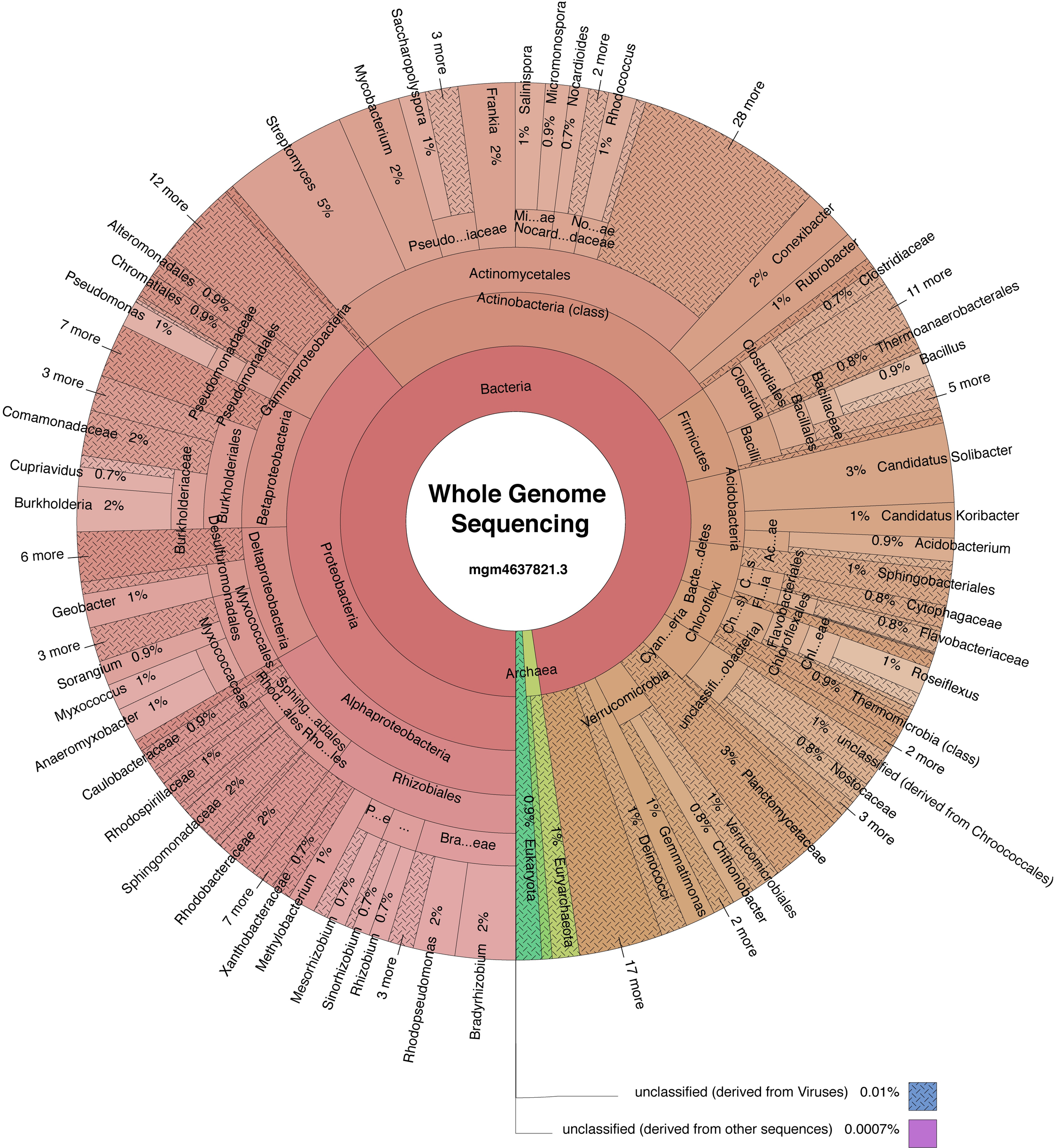

Supplement: S4 Fig — (TIF) [file pone.0228899.s004.tif]

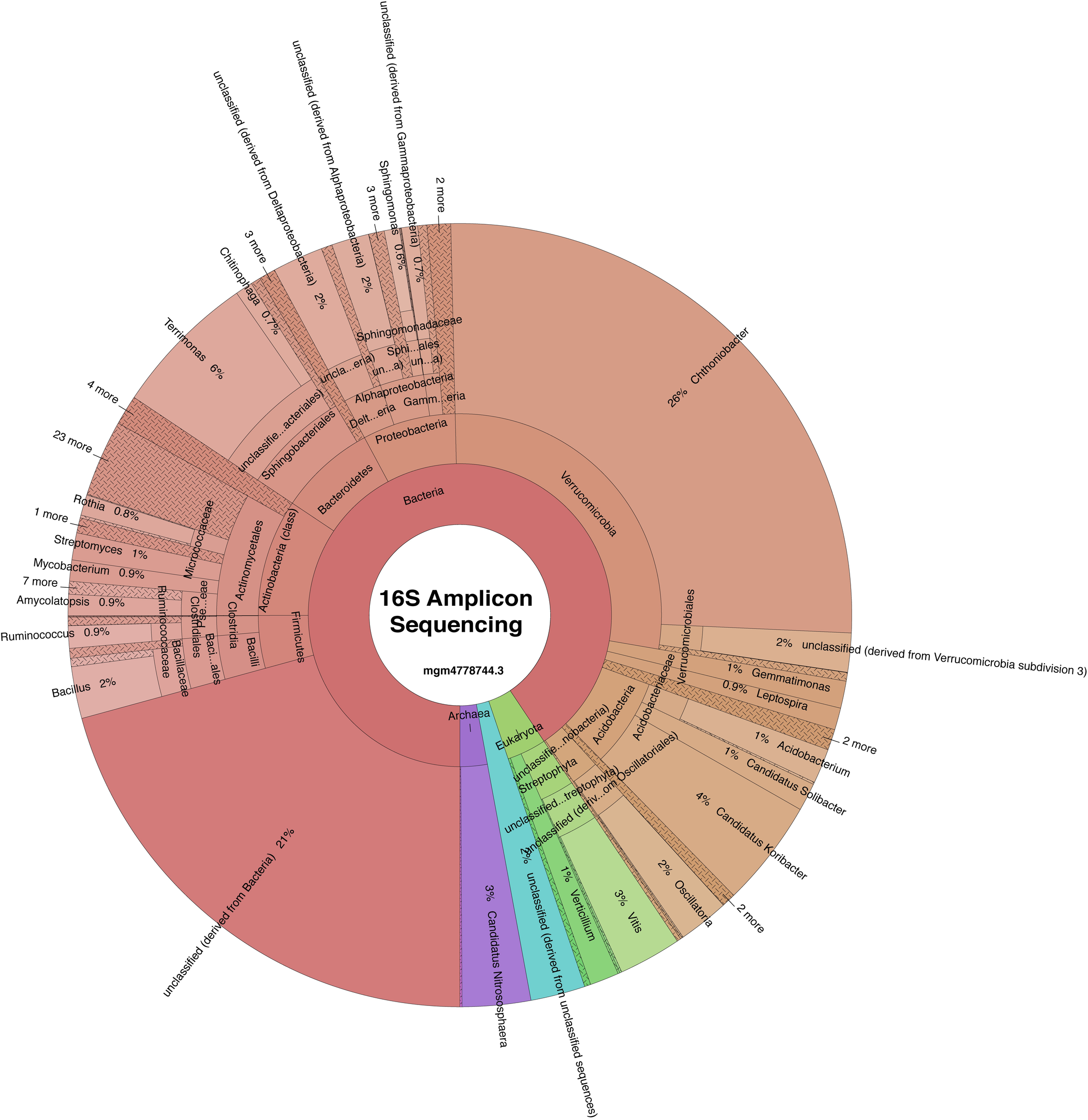

Supplement: S5 Fig — (TIF) [file pone.0228899.s005.tif]

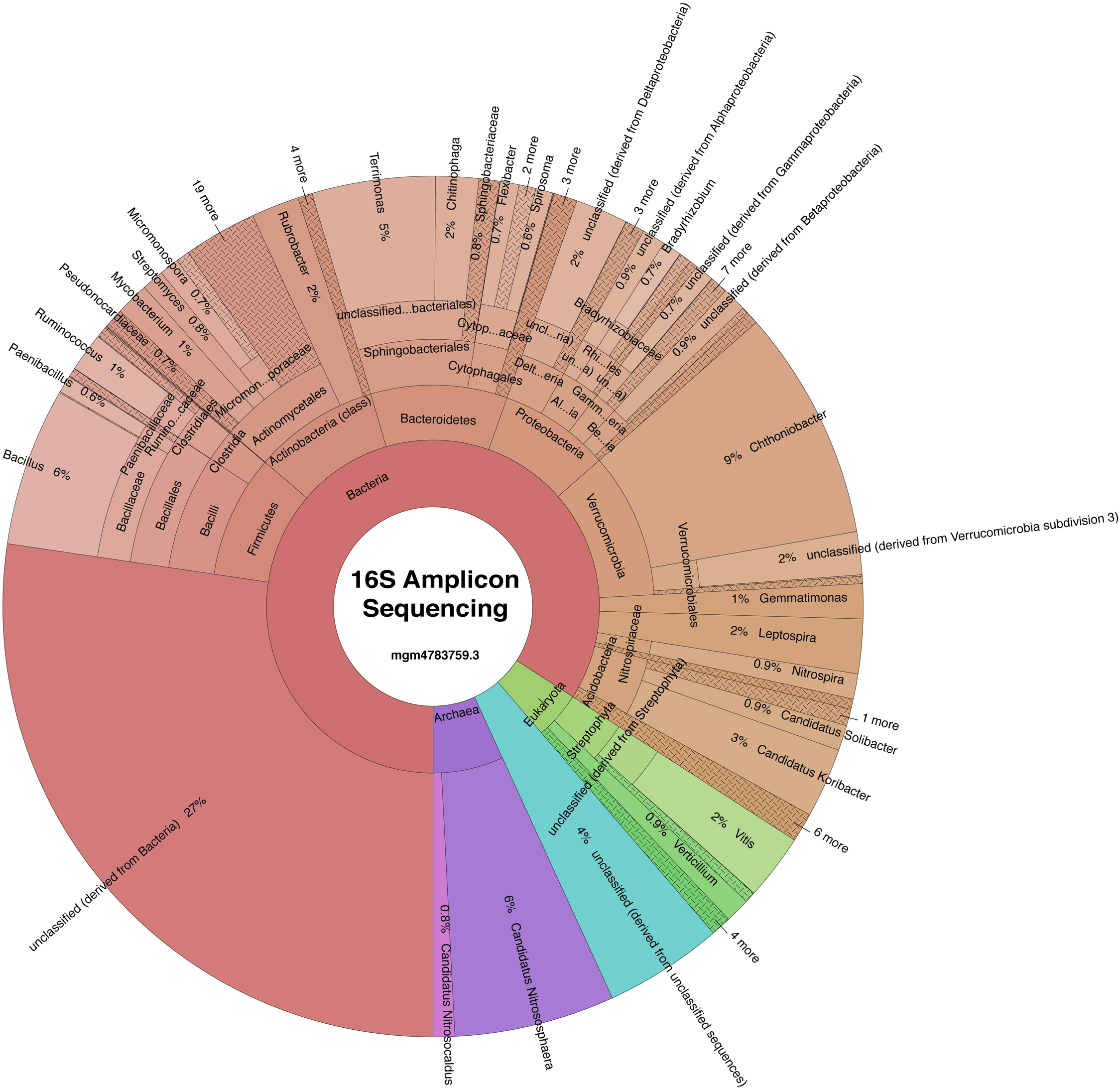

Supplement: S6 Fig — (TIF) [file pone.0228899.s006.tif]

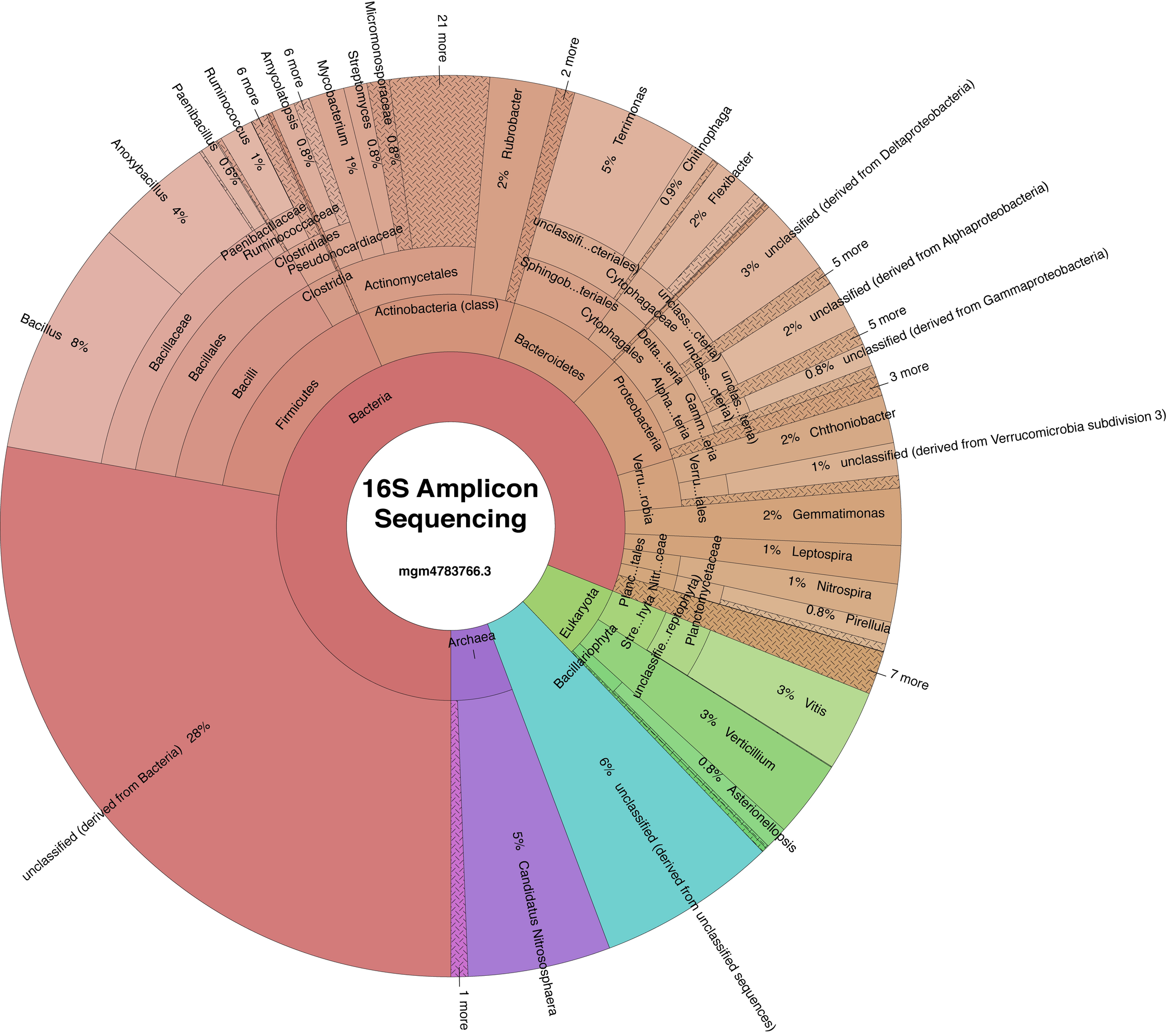

Supplement: S7 Fig — (TIF) [file pone.0228899.s007.tif]
